# Metabolically distinct roles of NAD synthetase and NAD kinase define the essentiality of NAD and NADP in *Mycobacterium tuberculosis*

**DOI:** 10.1128/mbio.00340-23

**Published:** 2023-06-23

**Authors:** Ritu Sharma, Travis E. Hartman, Tiago Beites, Jee-Hyun Kim, Hyungjin Eoh, Curtis A. Engelhart, Linnan Zhu, Daniel J. Wilson, Courtney C. Aldrich, Sabine Ehrt, Kyu Young Rhee, Dirk Schnappinger

**Affiliations:** 1 Department of Microbiology and Immunology, Weill Cornell Medical College, New York, New York, USA; 2 Department of Medicine, Weill Cornell Medical College, New York, New York, USA; 3 Center for Drug Design, Nils Hasselmo Hall, Minneapolis, Minnesota, USA; Washington University in St. Louis School of Medicine, St. Louis, Missouri, USA

**Keywords:** metabolism, NAD, NADPH, *Mycobacterium tuberculosis*, drug targets

## Abstract

Nicotinamide adenine dinucleotide (NAD) and its phosphorylated derivative (NADP) are essential cofactors that participate in hundreds of biochemical reactions and have emerged as therapeutic targets in cancer, metabolic disorders, neurodegenerative diseases, and infections, including tuberculosis. The biological basis for the essentiality of NAD(P) in most settings, however, remains experimentally unexplained. Here, we report that inactivation of the terminal enzyme of NAD synthesis, NAD synthetase (NadE), elicits markedly different metabolic and microbiologic effects than those of the terminal enzyme of NADP biosynthesis, NAD kinase (PpnK), in *Mycobacterium tuberculosis* (*Mtb*). Inactivation of NadE led to parallel reductions of both NAD and NADP pools and *Mtb* viability, while inactivation of PpnK selectively depleted NADP pools but only arrested growth. Inactivation of each enzyme was accompanied by metabolic changes that were specific for the affected enzyme and associated microbiological phenotype. Bacteriostatic levels of NAD depletion caused a compensatory remodeling of NAD-dependent metabolic pathways in the absence of an impact on NADH/NAD ratios, while bactericidal levels of NAD depletion resulted in a disruption of NADH/NAD ratios and inhibition of oxygen respiration. These findings reveal a previously unrecognized physiologic specificity associated with the essentiality of two evolutionarily ubiquitous cofactors.

**IMPORTANCE:**

The current course for cure of *Mycobacterium tuberculosis* (*Mtb*)—the etiologic agent of tuberculosis (TB)—infections is lengthy and requires multiple antibiotics. The development of shorter, simpler treatment regimens is, therefore, critical to the goal of eradicating TB. NadE, an enzyme required for the synthesis of the ubiquitous cofactor NAD, is essential for survival of *Mtb* and regarded as a promising drug target. However, the basis of this essentiality was not clear due to its role in the synthesis of both NAD and NADP. Here, we resolve this ambiguity through a combination of gene silencing and metabolomics. We specifically show that NADP deficiency is bacteriostatic, while NAD deficiency is bactericidal due to its role in *Mtb*’s respiratory capacity. These results argue for a prioritization of NAD biosynthesis inhibitors in anti-TB drug development.

## INTRODUCTION

In its oxidized form (NAD^+^), NAD serves as a signaling molecule (1) and is consumed as a substrate by various enzymes (2, 3). Conversion of NAD into its reduced (NADH) and phosphorylated derivatives [NADP(H)] results in two redox pairs, which are required as cofactors for over 1,500 hydride transfer reactions (4). In *Mycobacterium tuberculosis* (*Mtb*), NAD biosynthesis can occur by *de novo* synthesis and a salvage pathway that recycles NAD breakdown products (e.g., nicotinamide) (Supplementary Fig. 1a) (5, 6). Enzymes of either the *de novo* synthesis or the salvage pathway are thus dispensable. Enzymes required by both pathways, NadD (nicotinic acid mononucleotide adenylyltransferase) and NadE (NAD synthetase), however, are required for growth (5, 7, 8).

Inhibiting/depleting NadD or NadE is likely to result in a “metabolic catastrophe” due to the depletion of NAD(H), NADP(H), or both (9). Due to their pleiotropic effects, it becomes challenging to establish the chain of events triggered by deficient levels of these cofactors. In addition to NadD and NadE, the NAD kinase, PpnK, which catalyzes NADP biosynthesis, was also predicted to be required for *Mtb* growth (10). PpnK catalytic activity was experimentally shown (11) and its structure was solved (12, 13), but the consequences of its absence for *Mtb* metabolism are currently unknown.

Here, we resolve the roles of *Mtb* NadE in NAD(H) and NADP(H) biosynthesis, metabolism, and viability using conditional genetic knockdown strains of *Mtb* NadE and PpnK. We show that NADP(H) deficiency is bacteriostatic, while NAD(H) deficiency is bactericidal due to an impact in respiratory capacity.

## RESULTS

### Depletion of the NAD kinase PpnK is bacteriostatic in *Mtb*

**Fig 1:**
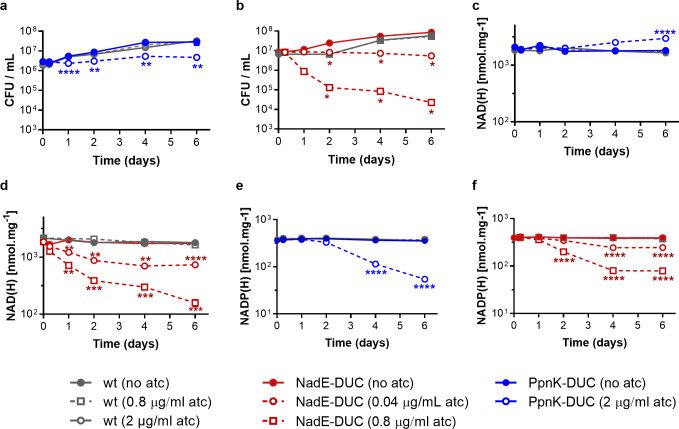
Impact of depleting NadE or PpnK on viability, NAD(H) and NADP(H) of *Mtb. Mtb* H37Rv wild-type (wt) and PpnK-DUC (**a**) or NadE-DUC (**b**) were grown on filters in 7H9 containing glucose as the sole carbon source with or without atc. CFUs were monitored for 6 days. Data are averages ± standard deviation (SD) of four (**a**) or two (**b**) filters and representative of at least two independent experiments. Intracellular NAD(H) (**c, d**) and NADP(H) levels (**e, f**) were measured over time in the presence (open symbols) and absence (closed symbols) of atc. Data are averages ± SD for triplicate filters and representative of at least two independent experiments. SDs are generally too small to extend beyond the symbols. ****P < 0.0001; ***P < 0.001; **P < 0.01; *P < 0.05 (unpaired *t*-test).

We previously showed that genetic depletion of NadE kills *Mtb* (14). NadE is required for the synthesis of NAD and NADP. It was thus not clear if death was due to a reduction in NAD, NADP, or both cofactors. To define the importance of NADP, we sought to selectively reduce pools of NADP but not NAD. To do so, we generated a PpnK knockdown mutant (PpnK-DUC) in which anhydrotetracycline (atc) induces the depletion PpnK by a dual-control (DUC) switch (14) that combines repression of *ppnK* transcription with controlled proteolysis of PpnK (Supplementary Fig. 1b through d). Depletion of *ppnK*, upon exposure to atc, prevented growth of *Mtb* on agar plates and in standard liquid media (Supplementary Fig. 1e through g) but did not reduce viability, as measured by colony forming units (CFU) counts, at atc concentrations as high as 2 µg/mL (Fig. 1a). This bacteriostatic phenotype was also observed with mixed carbon sources (Supplementary Fig. 1h). Depletion of NadE in NadE-DUC, in contrast, exhibited dose-dependent reductions in viability such that 0.04 µg/mL atc caused bacteriostasis, while 0.8 µg/mL atc killed NadE-DUC (Fig. 1b). The bactericidal effect upon NadE depletion and bacteriostatic effect upon PpnK depletion were also observed with glucose, acetic acid, or oleic acid as the primary carbon source (Supplementary Fig. 2a and b). Immunoblots demonstrated that NadE and PpnK were depleted with similar kinetics and to similar maximal degrees (Supplementary Fig. 3). The different effects caused by inactivation of PpnK or NadE were thus not due to more efficient depletion of NadE. These data suggest that similar levels of protein depletion result in death of NadE-DUC *Mtb* but persistence of PpnK-DUC *Mtb*.

### NAD(H) pools and NAD^+^/NADH correlate with bacterial death

To investigate the link between viability and cofactor depletion further, we next measured levels of NAD(H) and NADP(H) upon depletion of NadE or PpnK. We first analyzed the impact of different atc concentrations on NadE-DUC and found NAD levels to be reduced in a manner that was dose dependent and correlated with the extent of NadE depletion (Supplementary Fig. 4). Inactivation of PpnK caused a slight increase in NAD(H) pool size (Fig. 1c), while NadE-DUC using atc concentrations that induced bacterial stasis (0.04 µg/mL) and death (0.8 µg/mL) was associated with ~3-fold and ~10-fold reductions in total NAD(H) pools, respectively (Fig. 1d). Moreover, a fixed effects regression model demonstrated that both NAD(H) pool size (R^2^ = 0.98) and NAD^+^/NADH ratios (R^2^ = 0.53) correlated strongly with viability (as manifest at bactericidal levels of NadE depletion) compared with growth (as manifest at bacteriostatic levels of NadE depletion R^2^ = 0.33 and 0.31, respectively). Furthermore, depletion of PpnK and NadE lowered NADP(H) intracellular levels up to 6.5-fold and 5.0-fold, respectively (Fig. 1e and f). PpnK-DUC displayed little to no impact on ratios of either NAD^+^/NADH or NADP^+^/NADPH (Supplementary Fig. 5), indicating that a 6.5-fold reduction of NADP(H) was sufficient to cause stasis but not death of *Mtb*.

### Metabolomics define specific signatures for NAD(H) and NADP(H) deficiencies

NAD and NADP are annotated to participate in approximately 17% of the enzymatic reactions occurring in *Mtb* (15). It is thus not obvious by what, if any, specific mechanism(s) depletion of these cofactors might inhibit growth and how *Mtb* might compensate for the lack of NAD(H) and/or NADP(H). We sought to resolve this ambiguity by analyzing the changes that occur in the metabolome in response to silencing NadE or PpnK. For this, we grew *Mtb* using universally labeled ^13^C glucose as the sole source of carbon and analyzed how inactivation of NadE or PpnK affected the flow of carbon through the pathways of central carbon metabolism (CCM). We used 0.04 µg/mL of atc for NadE-DUC to avoid killing of *Mtb* and 2 µg/mL of atc for PpnK-DUC to achieve maximal depletion of PpnK.

**Fig 2:**
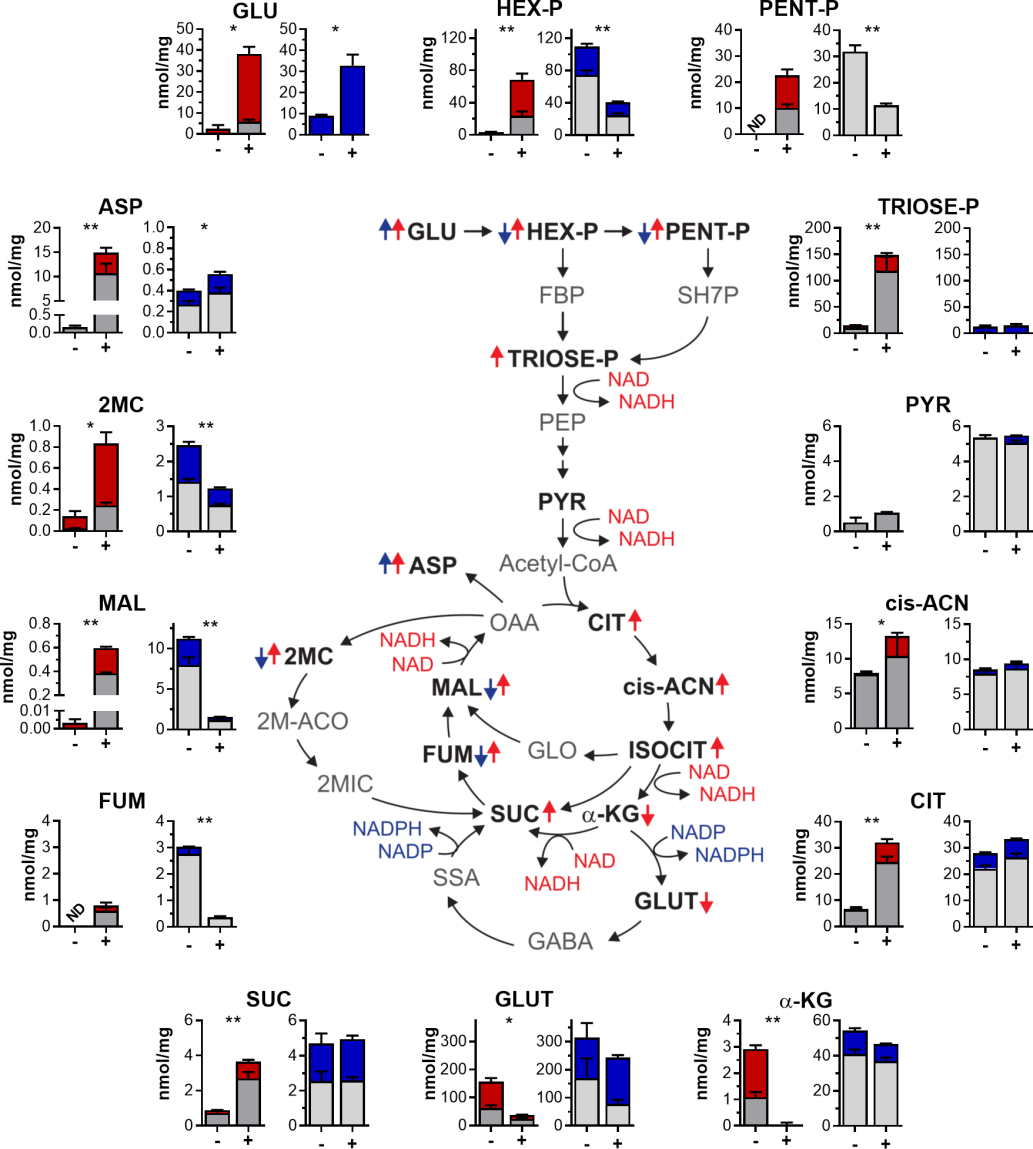
Impact of depleting NadE or PpnK on CCM metabolites. Intrabacterial pool sizes are shown for CCM metabolites isolated from NadE-DUC (red) or PpnK-DUC (blue), incubated in ^13^C glucose-containing media for 6 days. Bacteria were incubated with (+) or without (−) atc. Total bar heights indicate intrabacterial concentrations; the red or blue colored portion of each bar denotes the extent of ^13^C labeling following transfer to ^13^C glucose-containing 7H9. Data are averages ± SD for triplicate filters and representative of at least two independent experiments. ND (not detected) denotes that the metabolite level was below the limit of detection. Blue and red arrows specify metabolites that increase or decrease in response to depletion of PpnK or NadE, respectively. Abbreviations of metabolites that could not be quantified with and without atc are shown in gray. Abbreviations shown in bold indicated metabolites that could be measured in at least one condition. **P < 0.001; *P < 0.01 (unpaired *t* test; comparison ±atc within each strain). GLU, glucose; Hex-P, hexose phosphate; PENT-P, pentose 5-phosphates; TRIOSE-P, triose phosphates (glyceraldehyde 3-phosphate and dihydroxyacetone phosphate); PEP, phosphoenolpyruvate; PYR, pyruvate; αKG, α-ketoglutarate; CIT, (iso)citrate; cis-ACN, *cis*-aconitate; GLUT, glutamate; SSA, succinic semialdehyde; SUC, succinate; FUM, fumarate; MAL, malate; 2MC, 2 methyl citrate; ASP, aspartate; 2M-ACO, 2-methyl-*cis*-aconitate; 2MIC, 2-methylisocitrate; GLO, glyoxylate.

Many of the metabolites that changed after depletion of NadE to bacteriostatic levels of growth inhibition corresponded to NAD-dependent metabolic reactions. In glycolysis, the first enzyme requiring NAD is glyceraldehyde 3-phosphate dehydrogenase (GAPDH), which converts triose phosphates to phosphoenolpyruvate. Depletion of NadE caused accumulation of the four glycolytic metabolites preceding the GAPDH reaction (Fig. 2). Consistent with its role as a respiratory cofactor used to sustain canonical tricarboxylic acid (TCA) cycle activity, we observed altered levels of TCA cycle intermediates following inactivation of NadE (Fig. 2). Accordingly, changes in malate, fumarate, and succinate were among the most pronounced following NAD depletion (Supplementary Fig. 6).

Depletion of PpnK significantly affected the levels of several CCM metabolites, most of which were depleted (Fig. 2). These included metabolites of NADP(H)-dependent reactions, but many metabolites were neither substrates nor products of known NADP-dependent metabolic reactions. Depletion of PpnK thus appears to affect CCM indirectly via mechanisms that remain to be elucidated. Mechanistic ambiguities notwithstanding, these data demonstrate that PpnK and NadE play distinct and largely nonoverlapping roles in CCM.

### NAD(H) deficiency reduces respiratory capacity

**Fig 3:**
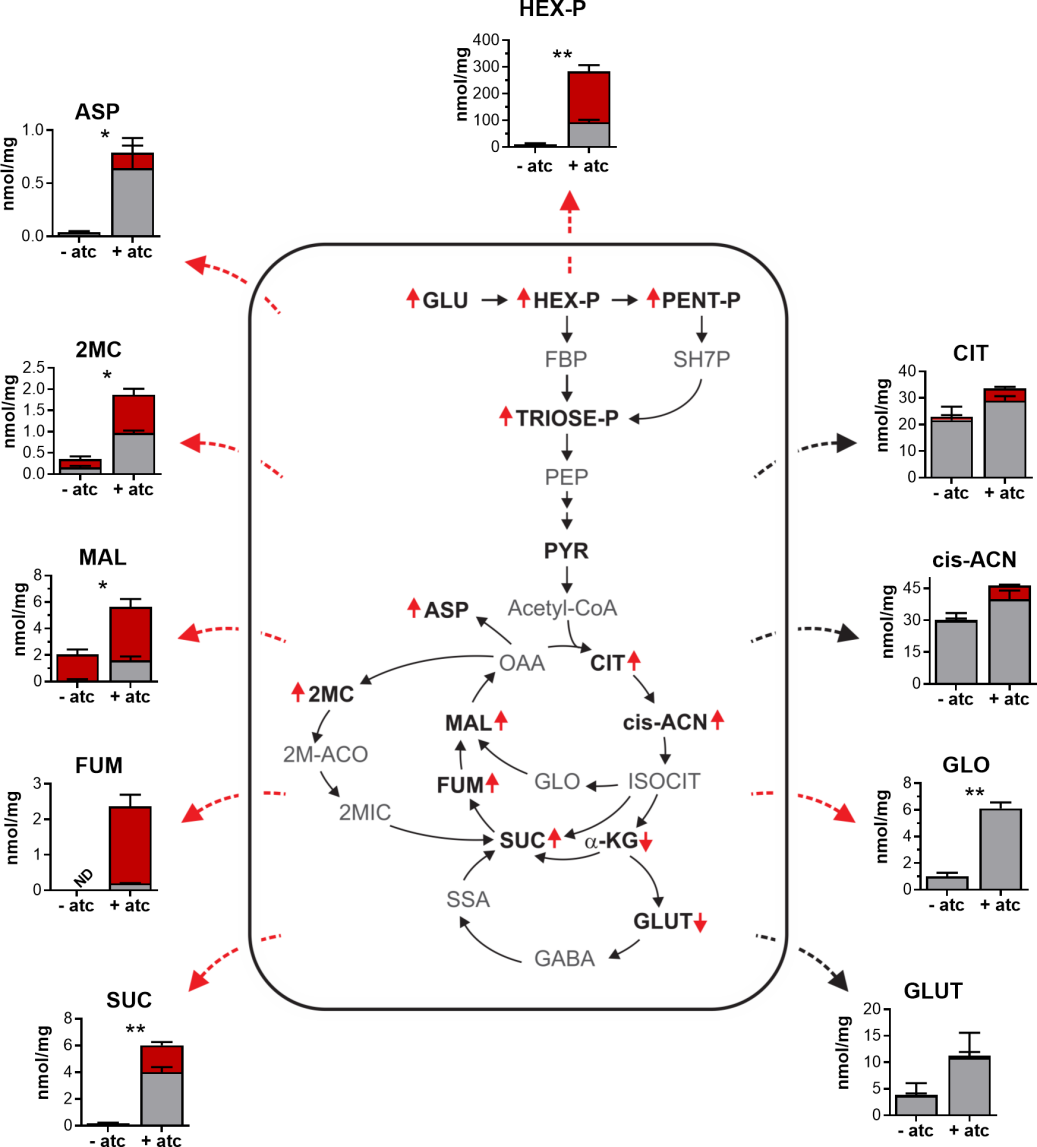
Metabolites accumulating in the media upon NadE depletion. NadE-DUC was incubated in ^13^C glucose-containing 7H9 media after preadaptation on 7H9 glucose agar plates. Metabolites in the media were measured at day 6 with 0.04 µg/mL of atc (+atc) or without atc (−atc). Total bar heights indicate the concentration of the metabolite in the pool media, and the red-colored portion for each bar denotes the extent of ^13^C labeling achieved following transfer to ^13^C glucose. Red-dotted arrows indicate metabolite whose amount in media increased upon NadE depletion. Solid red arrows specify intrabacterial metabolites that increase or decrease in response to depletion of NadE, from Fig. 2. Data are averages ± SD for triplicate filters and representative of at least two independent experiments. ND denotes that the metabolite level was below the limit of detection. Abbreviations of metabolites that could not be quantified with and without atc are shown in gray. Abbreviations shown in bold indicated metabolites that could be measured in at least one condition. **P < 0.001; *P < 0.01 (unpaired *t* test; comparison ±atc within each strain). GLU, glucose; Hex-P, hexose phosphate; PENT-P, pentose 5-phosphates; TRIOSE-P, triose phosphates (glyceraldehyde 3-phosphate and dihydroxyacetone phosphate); PEP, phosphoenolpyruvate; PYR, pyruvate; αKG, α-ketoglutarate; CIT, (iso)citrate; cis-ACN, *cis*-aconitate; GLUT, glutamate; SSA, succinic semialdehyde; SUC, succinate; FUM, fumarate; MAL, malate; 2MC, 2 methyl citrate; ASP, aspartate; 2M-ACO, 2-methyl-*cis*-aconitate; 2MIC, 2-methylisocitrate; GLO, glyoxylate.

**Fig 4:**
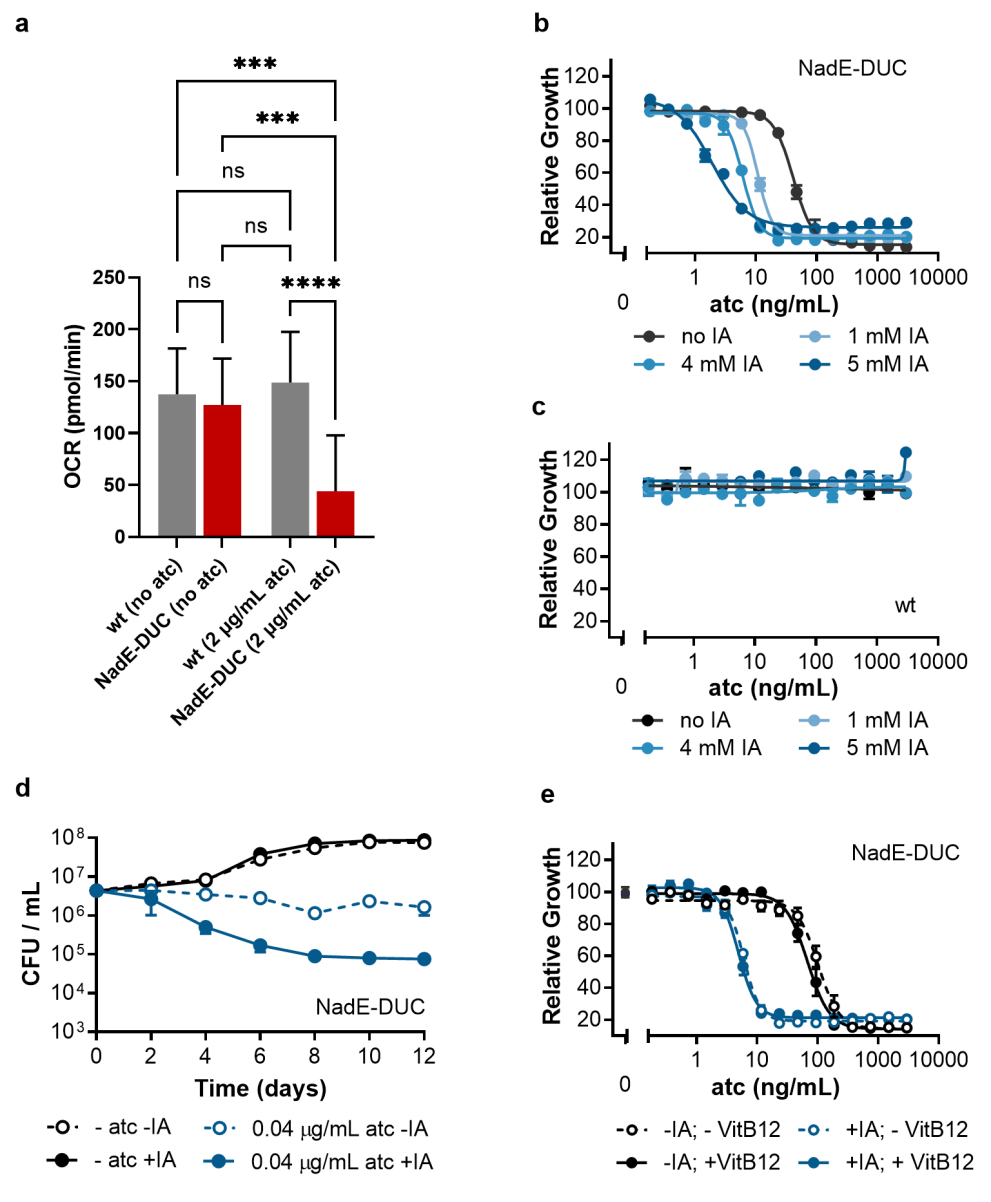
Impact of NadE-depleted *Mtb* in oxygen consumption rate and susceptibility to itaconic acid (IA). (**a**) *Mtb* H37Rv and NadE-DUC were grown in 7H9 containing glucose as the sole carbon source, then switched to media with or without atc for 48 hours. OCR data at 48 hours represent averages of 12 replicates ± standard error of the mean (SEM) of a representative experiment of three independent experiments. ****P < 0.0001; ***P < 0.001; ns, not significant one-way followed by post hoc test (Tukey test; GraphPad Prism). Atc dose–responses for NadE-DUC (**b**) and H37Rv (c) with and without IA and with and without vitamin B12 (VitB12) (**d, e**) Impact of IA on viability of NadE-DUC. Values are averages ± SD of six (**b**, **c**, **d**) or three samples and representative of at least two independent experiments.

The changes in TCA cycle activity accompanying NadE depletion suggest that depletion of NAD pools may impose an additional limitation on *Mtb*’s total capacity to turnover NADH and regenerate NAD^+^, and thereby inhibit other NAD(H)-dependent reactions. Consistent with such a limitation for NADH-based turnover of reducing equivalents, we observed both intra- and extra-cellular accumulations of intermediates of the reductive arm of the TCA cycle under bacteriostatic levels of NadE depletion (Fig. 3). The consequence of this accumulation was a disruption of aerobic respiratory chain capacity driven by the depletion of NAD(H). This is evidenced by the threefold reduced oxygen consumption rate (OCR) observed when NAD(H) was depleted in the NadE-DUC strain, while OCR by wild-type *Mtb* remained unaffected (Fig. 4a). Also, the observed effect in OCR upon NadE depletion did not stem from altered expression of oxidative phosphorylation enzymes encoding genes (Supplementary Fig. 7).

### Bacteriostatic levels of NadE trigger a metabolic adaptive activation of the glyoxylate shunt

To further investigate the basis for these accumulations, we compared the ^13^C labeling patterns of TCA cycle intermediates in wild-type and NadE-depleted *Mtb*. We specifically hypothesized that depletion of NAD pools could trigger either an adaptive reversal of TCA cycle flux or activation of the glyoxylate shunt. To differentiate between these two possibilities, we sought evidence of reductive TCA cycle activity, as manifest by an accumulation of M + 3 isotopologs (16). Failing to observe a uniform enrichment of M + 3 isotopologs (Supplementary Fig. 8), we hypothesized that NAD depletion triggered an adaptive activation of the glyoxylate shunt. To test this hypothesis, we measured the impact of inhibiting the glyoxylate shunt enzyme, isocitrate lyase (ICL), on *Mtb*’s sensitivity to NAD depletion. We found the ICL inhibitor, itaconic acid, decreased the MIC of atc for NadE-DUC but not wild-type *Mtb*, up to ~20-fold (Fig. 4b and c) and sensitized NadE-DUC to killing by incomplete depletion of NadE (Fig. 4d).

*Mtb*’s ICL is bifunctional isocitrate and methylisocitrate lyases and participates in both the glyoxylate shunt and methylcitrate cycle (17). However, vitamin B12, a cofactor for the methylmalonyl-CoA pathway, which can bypass the methylcitrate cycle but not the glyoxylate shunt (18), did not change the activity of itaconic acid on NadE-DUC (Fig. 4e). Itaconic acid thus affected NadE-DUC by inhibiting the glyoxylate cycle and not the methylcitrate cycle. Inhibitors of RNA polymerase (rifampicin and fidaxomicin), the ribosome (chloramphenicol and linezolid), energy metabolism (bedaquiline, nigericin, and valinomycin), and cell envelope biosynthesis (cerulenin, ethionamide, isoniazid, and BTZ04) did not increase sensitivity of NadE-DUC to depletion of NadE as much as itaconic acid (Supplementary Fig. 9). Depletion of PpnK did not alter the sensitivity to the same panel of inhibitors (Supplementary Fig. 10). Taken together, these experiments demonstrate that bacteriostatic levels of NadE depletion trigger an adaptive metabolic activation of the glyoxylate shunt, while bactericidal levels of depletion compromise *Mtb*’s ability to maintain NADH/NAD homeostasis.

## DISCUSSION

A fundamental, but unanswered, question is whether the essentiality of NAD(H) and NADP(H) is a consequence of specific reactions they serve, their aggregate participation in numerous biochemical reactions, or their role in redox homeostasis. The answer to this question is of basic biological and therapeutic importance because it addresses the biochemical organization of metabolic networks and novel potential vulnerabilities. By combining a conditional gene silencing approach with microbiological, biochemical, and metabolomic assays to determine the consequences of inactivating NAD synthase (NadE) to bacteriostatic and bactericidal levels of NAD depletion and comparing them with inactivation of NAD kinase (PpnK), our work provides mechanistic insight into the essentiality of NadE in *Mtb*. We specifically demonstrate that the bactericidal essentiality of NadE is tied to a loss of NAD(H), rather than NADP(H), dependent activities, and occurs following an approximately 3- to 10-fold depletion of total NAD(H) pools. We further show that bacteriostasis is associated with a selective impact on NAD(H)-dependent metabolic reactions, while bacterial death is associated with a loss of NAD/NADH redox homeostasis. Our studies further show that the metabolic changes preceding this loss of redox homeostasis reflect an adaptive response to a functional decrease in aerobic respiratory chain capacity that implicates the respiratory chain as the primary functional target of NAD(H) depletion and may help explain the efficient sterilization of hypoxia-adapted, nonreplicating *Mtb* that resulted from NadE depletion (14).

Despite its annotated participation in hundreds of biochemical reactions, knowledge of the fundamental physiological role(s) of NAD(H) remains surprisingly incomplete. Our studies help to close this gap with the experimental discovery of a previously unrecognized physiological hierarchy of NAD(H)-dependent activities in the human-adapted pathogen, *Mtb*. We specifically show that the essentiality of NAD(H) is mediated in large part by its role as a respiratory cofactor, depletion of which initially results in an adaptive remodeling of its metabolism but ultimately fails due to an inability to maintain redox homeostasis. These studies thus highlight a previously underappreciated function of NAD(H) and vulnerability of *Mtb*—respiratory capacity. Analogous to the concept of total lung capacity in humans, we define respiratory capacity as a static measure of an organism’s maximum potential respiratory activity that, when compromised, can impair fitness and survival like humans with emphysema. The discovery of NAD(H) as a molecular determinant of bacterial respiratory capacity not only expands our understanding of a validated and attractive target but also identifies novel strategies that exploit this vulnerability, including approaches with the potential to create the functional equivalent of emphysema in bacteria. In a broader biological context, these studies help to dissociate biochemical ubiquity of a molecule from its physiological specificity.

## MATERIALS AND METHODS

### Strains, media, and culture conditions

*Mtb* H37Rv wt, NadE-DUC, and PpnK-DUC strains were grown in Middlebrook 7H9 liquid media supplemented with 0.5% bovine serum albumin fraction V (BSA), 0.2% glucose, 0.2% glycerol, 0.085% NaCl, and 0.05% tyloxapol without shaking in 5% CO_2_ at 37°C or on Middlebrook 7H10 agar containing OADC (Becton Dickinson and company) and 0.5% glycerol. For single carbon source media, 7H9 was supplemented with 0.5% fatty acid-free BSA, 0.085% NaCl, and the following carbon sources: 0.2% glucose, 0.2% acetate, or 0.6% oleic acid (added as sodium oleate). When appropriate, hygromycin B (50 µg/mL), kanamycin (25 µg/mL), nourseothricin (25 µg/mL), and zeocin (25 µg/mL) were used. Atc was added at the indicated concentrations and replenished at half the initial concentration in liquid cultures every 3–4 days for growth curves. For survival assays, bacterial culture samples were taken from growth curve cultures at the time points indicated and plated for CFU.

### Mutant strain construction and essentiality test

Construction of NadE-DUC has previously been reported (14). PpnK-DUC was constructed in a similar manner. *Mtb* H37Rv was transformed with a plasmid expressing *ppnK* under the control of its native promoter (spanning 652 bp upstream of *ppnK*) that integrates into the phage attL5 site in the *Mtb* genome. In this *ppnK* merodiploid strain, the native copy of *ppnK* was deleted as previously described (19, 20). After confirming deletion of the native copy of *ppnK* by Southern blot, replacement transformations of the attL5 insets were performed to generate PpnK-DUC. In the PpnK-DUC mutant, *ppnK* was expressed under the control of a TetOFF promoter and with a C-terminal DAS+4 tag. A plasmid in the phage tweety site was introduced, which allowed the inducible expression of *SspB* (14). Essentiality test of *ppnK* for *in vitro* growth was done by transforming the *Mtb ΔppnK::PppnK* mutant with zeocin resistance plasmids expressing *ppnK* or vector control. The transformants were selected on 7H10 agar containing zeocin.

### Immunoblot analysis of cytosolic proteins

Protein extracts were prepared from bacterial pellets from NadE-DUC, PpnK-DUC, and *Mtb* H37Rv cultures or bacteria-laden filters at indicated time points in specified media. Briefly, cultures were washed with phosphate buffered saline (PBS) with 0.05% Tween 80 and resuspended in 500 µL PBS, 1× protease inhibitor cocktail (Roche). Cells were lysed by bead beating three times at 4,500 rpm for 30 seconds with 0.1-mm Zirconia/Silica beads. Beads and cell walls were removed through centrifugation (11,000 × *g*/10 minutes, 4°C), and the supernatant was filtered through a 0.2-µM Spin X column (Corning). Lysate protein concentrations were determined using a DC Protein Assay Kit (Bio-Rad). For immunoblots, 25-µg protein extracts were separated by SDS-PAGE, transferred to nitrocellulose membranes, and probed overnight with antisera against nadE and ppnK (both at 1:1,000 dilution) or PrcB (1:15,000 dilution). Proteins were detected using the Odyssey Infrared Imaging System (LI-COR Biosciences).

### *Mtb* growth on filters and metabolite extraction

For *Mtb* strains NadE-DUC, PpnK-DUC, and *Mtb* H37Rv, cultures were preadapted in 7H9 broth supplemented with 0.2% glucose, 0.05% tyloxapol, 0.5 g/L BSA, and 0.085% NaCl. One milliliter of culture at optical density at 580 nm (OD_580_) of 1.0 was filtered through a nitrocellulose membrane (0.22 µM; Millipore GSWP 02500) (21, 22) and incubated on 7H9 agar (containing 15% agar, 0.2% glucose, 0.5 g/L BSA, and 0.085% NaCl) at 37°C for 7 days to reach the midlogarithmic phase of growth. *Mtb*-laden filters were then transferred onto a “swimming pool” made from inverted 15-mL conical tubes filled with chemically identical medium containing fresh ^12^C glucose or [U-^13^C] glucose-supplemented 7H9 (containing 0.5 g/L BSA and 0.085% NaCl). *Mtb*-laden filters were metabolically quenched by plunging filters into a mixture of acetonitrile/methanol/H_2_O (40:40:20) precooled to −20°C; metabolites were extracted by mechanical lysis with 0.1-mm Zirconia beads in a Precellys tissue homogenizer for 3 minutes (6,500 rpm) twice under continuous cooling at or below 2°C. Lysates were clarified by centrifugation and then filtered across a 0.22-µM filter. Residual protein content of metabolite extracts (Pierce BCA Protein Assay kit; Thermo Scientific) was determined to normalize samples to cell biomass. To analyze secreted metabolites, 1 mL of swimming pool medium was extracted from each pool and passed through a Spin X column (23). All data obtained by metabolomics were calculated from the average of independent triplicates

### Metabolomics with liquid chromatography-mass spectrometry

Metabolite quantification was accomplished using an Agilent 1200 liquid chromatography (LC) system coupled via electrospray ionization to either an Agilent 6230 TOF or 6520 Q-TOF. Metabolites were separated by LC on a Cogent Diamond Hydride type C column (MicroSolv Technology, Eatontown, NJ, USA) as previously described (24). Putative m/z values were identified by exact mass, with a mass tolerance of <0.005 Da. Reported metabolites were verified and quantified using a calibration curve generated with an authentic chemical standard.

### Isotopolog analysis using isotope-labeled carbon sources

The extent of isotopic labeling for metabolites was determined by dividing the summed peak height ion intensities of all labeled isotopolog species by the ion intensity of both labeled and unlabeled species, expressed in percentage. Label-specific ion counts were corrected for naturally occurring ^13^C species (i.e., [M+1] and [M+2]). The relative abundance of each isotopically labeled species was determined by dividing the peak height ion intensity of each isotopic form (corrected for naturally occurring ^13^C species as above) by the summed peak height ion intensity of all labeled species. Ion intensities were converted into molar abundances by using standard curves generated by the addition of chemical standards and serial dilution of samples to establish the collinearity of ion intensity and molar abundance.

### OCR measurements using the Seahorse XFe96 analyzer

OCR was measured as described in reference 25. Briefly, *Mtb* H37Rv and NadE-DUC strains were grown in 7H9 containing glucose as the sole carbon source, then switched to media with or without atc for 48 hours. Cells were harvested and ~5 × 107 CFU/mL cells were added into Agilent Seahorse cell culture plates that had been coated with 22.4 µg/mL Cell Tak (Corning) and briefly centrifuged at 4,000 rpf. The plates were then assayed using a Seahorse XFe96 analyzer (Agilent) programmed to assay four basal measurements followed by a decoupling step to measure spare respiratory capacity using the uncoupler carbonyl cyanide m-chlorophenyl hydrazone. Agilent Wave Desktop software was used to analyze the resulting data, which was exported to Microsoft Excel for averaging and GraphPad Prism 9.4 for figures.

### Quantitative PCR

NadE-DUC was grown in 7H9 liquid medium until exponential phase (OD_580_ of 0.5). Samples were harvested by adding one volume of guanidine thiocyanate buffer. RNA extraction was performed with TRIzol reagent (Invitrogen) and “Quick-RNA Miniprep Kit” (Zymo Research), following the manufacturer’s instructions. Samples were cleaned from genomic DNA using the “Turbo DNA-free kit” (Invitrogen). cDNA was synthesized from 500 ng of RNA using M-MuLV Reverse Transcriptase (New England BioLabs). LightCycler 480 SYBR Green I Master (Roche) in the LightCycler 480 System (Roche) was used to perform quantitative PCR. The software RealTimeDesign (Biosearch Technologies) was used to design primers and probes, which were ordered from TIB Molbiol, LLC. Probes for genes of interest were labeled with 5′ 6-FAM/3′ BHQ1, and the probe for the reference gene sigA was labeled with 5′ LC670.

### Cell viability test

Cell viability of *Mtb* H37Rv, NadE-DUC, and PpnK-DUC with or without atc treatment was determined using liquid cultures manipulated under experimentally identical conditions as filter-grown cultures used for metabolomic profiling, which we had previously demonstrated to be microbiologically similar. CFUs were determined by plating on 7H11 agar with supplements (0.2% glycerol, 0.2% glucose, 0.5 g/L BSA, and 0.085% NaCl).

### Measurement of NAD(H) and NADP(P) cofactor levels

*Mtb* H37Rv, NadE-DUC, and PpnK-DUC cultures were grown on filters as previously described. NAD and NADH, and NADP and NADP(H) concentrations were measured using a FluroNAD/NADH detection kit (Cell Technology). Each condition was measured in triplicate, and each experiment was performed twice.

### Itaconic acid sensitivity assay

*Mtb* H37Rv, NadE-DUC, and PpnK-DUC cultures were preadapted in specific, single-carbon 7H9 media and diluted to an OD_580_ of 0.02. Bacteria were then exposed to different doses of itaconic acid. OD_580_ was recorded after 12 days and normalized to the corresponding strains without treatment. Minimum inhibitory concentration is defined as the lowest concentration of a drug at which bacterial growth was inhibited at least 90%, as compared with the control containing no antimicrobial compounds.

### Inhibitor panel susceptibility assay

*Mtb* NadE-DUC culture was preadapted in glucose 7H9 medium (0.5% BSA, 0.085% NaCl, 0.2% glucose, and 0.05% tyloxapol) and diluted to an OD_580_ of 0.01 at various concentrations of atc (0, 125, 250, and 500 ng/mL). Diluted cultures were added to assay-ready 384-well plates for a final assay volume of 50 µL per well. Assay-ready plates contained inhibitor doses in dimethyl sulfoxide(DMSO) in a fourfold, 11-point dilution series, for a DMSO concentration of 1% following addition of diluted cultures. Plates were prepared directly from 100% DMSO stocks using an HP D300e Digital Dispenser or were stamped from a 10% DMSO master plate using a Tecan Freedom EVO 150 equipped with a MultiChannel Arm and a 384-well head adapter. Each inhibitor series was tested in duplicate for each atc condition. Plates were incubated at 37°C and 5% CO_2_ in a humid incubator, and OD_580_ was recorded after 14 or 16 days. The mean of the optical density data was normalized to the corresponding atc condition’s no-inhibitor control.

### Statistical analysis

Analyses were performed by the unpaired *t* test and analysis of variance test. A *P*-value of less than 0.05 was considered statistically significant. Curve pair correlations were determined using a fit fixed effects regression model on the pairs of the two quantities (both log-transformed) collected over time. Measure of goodness of fit, the overall R^2^, is reported for each condition and in relation to a squared correlation such that values closer to 1 reflect a stronger linear relationship between the two variables (or curves) over time.

## Data Availability

The data that support the findings of this study are available either within this article and its supplementary information files or upon reasonable request to the corresponding author.

## SUPPLEMENTAL MATERIAL

Supplemental Data mbio.00340-23-s0001.pdf Fig. S1 to S10.

ASM does not own the copyrights to Supplemental Material that may be linked to, or accessed through, an article. The authors have granted ASM a non-exclusive, world-wide license to publish the Supplemental Material files. Please contact the corresponding author directly for reuse.
