# Supplementary material for: Metabolically distinct roles of NAD synthetase and NAD kinase define the essentiality of NAD and NADP in Mycobacterium tuberculosis
Source: mBio. 2023 Jun 23;14(4):e00340-23. doi: 10.1128/mbio.00340-23 (PMC10470730; doi:10.1128/mbio.00340-23)
Supplement: Supplemental Data — Fig. S1 to S10. [file mbio.00340-23-s0001.pdf]

Metabolically distinct roles of NAD synthetase and NAD kinase define the essentiality of NAD and NADP in *Mycobacterium tuberculosis*

Ritu Sharma[1], Travis Hartman[3], Tiago Beites[1], Jee-Hyun Kim[1], Hyungjin Eoh[2,3], Curtis A. Engelhart[1], Linnan Zhu[1], Myung Hee Lee[3], Daniel J. Wilson[4], Courtney C. Aldrich[4], Sabine Ehrt[1], Kyu Rhee[3]*, Dirk Schnappinger[1]*

[1] Department of Microbiology and Immunology, Weill Cornell Medical College, New York, NY, USA.

[2] Current Address: Department of Molecular Microbiology and Immunology, Keck School of Medicine, University of Southern California, USA

[3] Department of Medicine, Weill Cornell Medical College, New York, NY, USA.

[4] Department of Medicinal Chemistry, University of Minnesota, 308 Harvard Street SE, 8-174 WDH, Minneapolis, Minnesota 55455, United States

Correspondence should be addressed to D.S. (dis2003@med.cornell.edu) or K.R (kyr9001@med.cornell.edu).

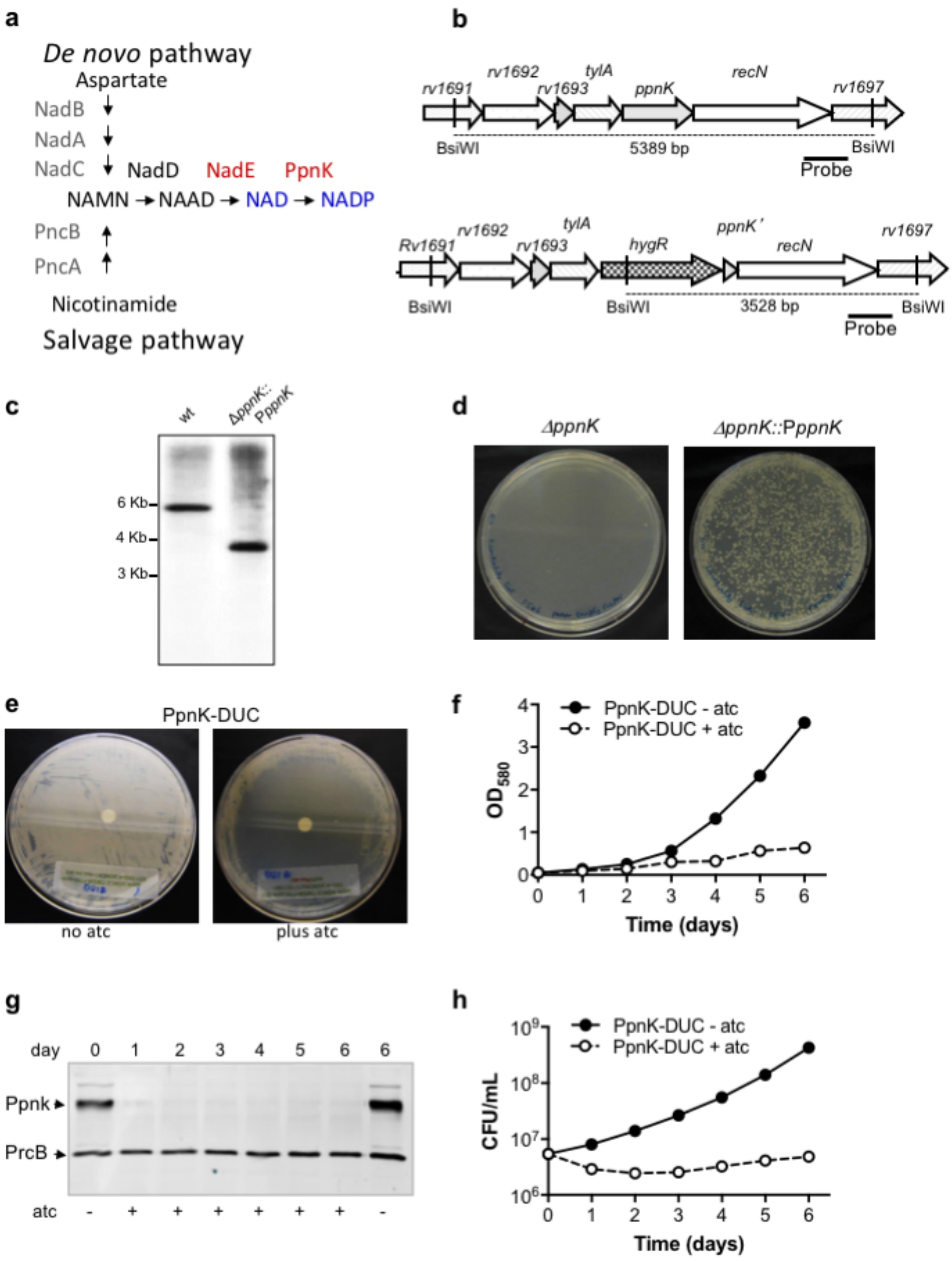

Supplementary Fig. 1. Construction and initial characterization of *Mtb* PpnK-DUC. **a,** Biosynthesis of NAD and NADP. The de novo and salvage pathways for the biosynthesis of NAD converge on the final two steps, which are catalyzed by NadD and NadE. Silencing either is expected to impact not only the levels of NAD but also of NADP. PpnK is the only *Mtb* enzyme known to generate NADP from NAD. **b,** Genomic organization of the *ppnK* region in H37Rv and Δ*ppnK*::P*ppnK.* To construct PpnK-DUC we first generated a merodiploid strain by integrating pGMCS-0X-PppnK (which contains *ppnK* and its native upstream region) into the attL5 site. The original chromosomal copy of *ppnK* gene was then replaced with a hygromycin cassette by homologous recombination. This resulted in *Mtb*

Δ*ppnK*::P*ppnK*. **c,** Confirmation of Δ*ppnK*::P*ppnK* by southern blotting. Genomic DNA from H37Rv and *ΔppnK::PppnK* were digested with BsiWI and probed with the DNA fragment indicated in (b). **d**, Essentiality of *ppnK*. To test essentiality of ppnK, the plasmid pGMCS-0X-PppnK of *ΔppnK::PppnK* was replaced either with an "empty" plasmid (left) or another PpnK expression plasmid (right). Only plasmids containing *ppnK* yielded colonies demonstrating that *ppnK* is required for growth on solid agar plates. **e,** Growth of PpnK-DUC on 7H11 agar. To generate the dual-control (DUC) mutant, the pGMCS-0X-PppnK plasmid located at the attL5 site of *ΔppnK:PppnK* was replaced with a plasmid that contained (i) DAS-tagged *ppnK* transcribed by the tet-operator containing promoter P750, and (ii) a gene enconding a reverse tet repressor transcribed by a constitutive promoter. In addition, the mutant was transformed with a plasmid that integrates in the tweety phage attachment site and expresses the SspB adaptor protein under the control of wt tet repressor to generate PpnK-DUC. To test the regulation, PpnK-DUC was plated on 7H11 plates either with an atc free (left) or an atc-containing paper (right) disc. **f,** Growth of PpnK-DUC in 7H9 liquid media. PpnK-DUC was inoculated at an initial OD of 0.01 and cultivated without or with 2 µg/mL atc. **g,** Immunoblot analysis of PpnK. PpnK-DUC was grown in 7H9 media and treated with 2 µg/mL atc. Samples were collected from cells that were treated with atc as well as from untreated cells from 1-6 days. 25 µg of total protein extract was resolved on 10% SDS PAGE gels and analyzed with PpnK-specific antisera. Antisera against PrcB was used to detect the loading control. **h,** Impact of PpnK depletion on survival in 7H9 medium containing glucose, glycerol and fatty acids as carbon sources. Survival was quantified by CFU enumeration.

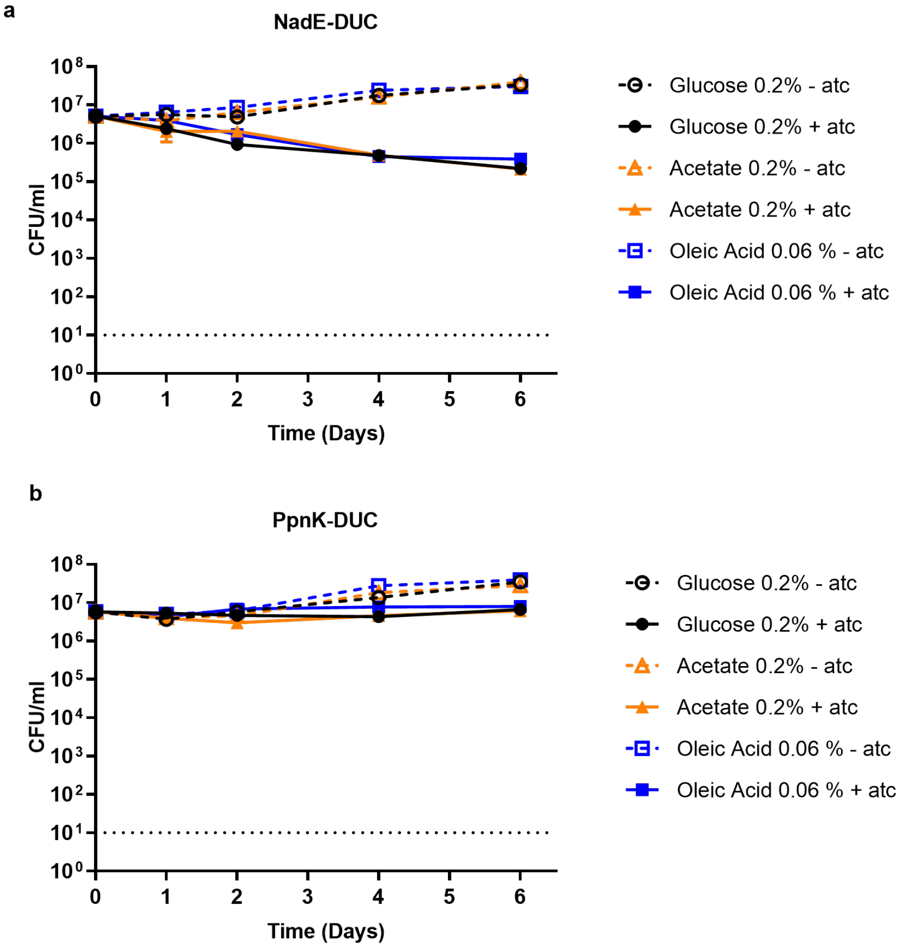

Supplementary Fig 2. Impact of NadE or PpnK depletion in media with single carbon sources. The strains NadE-DUC (-atc or + 0.8 µg/ml atc) (**a**) and PpnK-DUC (-atc or + 2 µg/ml atc) (**b**) were cultured in 7H9 supplemented with fatty acid-free bovine serum albumin, NaCl and glucose 0.2 %, acetate 0.2 % or sodium oleate 0.06 % as single carbon sources. CFUs were counted after outgrowth in 7H11 plates.

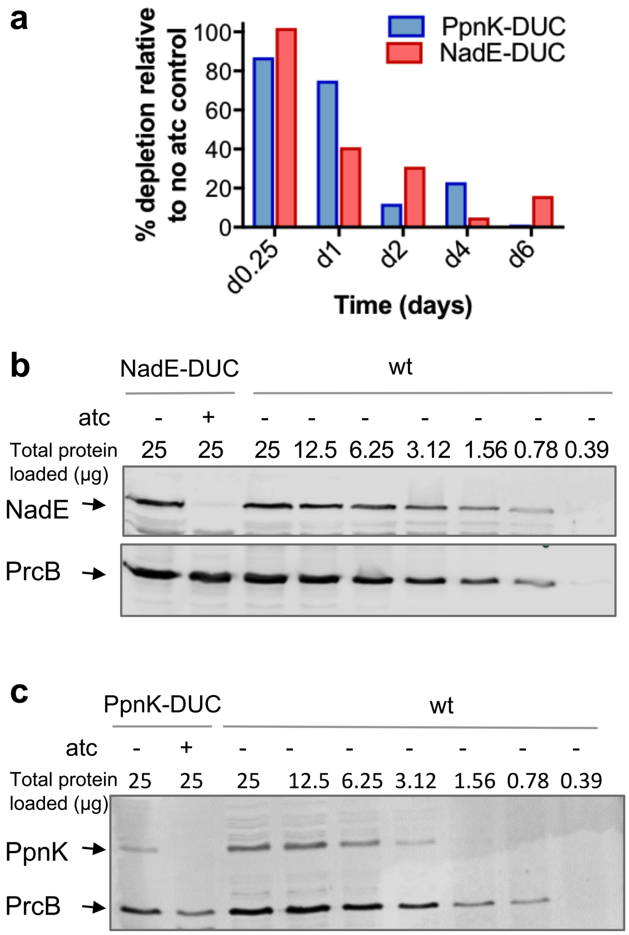

Supplementary Fig. 3. Kinetics and extent of NadE and PpnK depletion in NadE-DUC and PpnK-DUC. a, Kinetics of depletion. Bacteria were grown in 7H9 containing glucose as the single carbon source in the presence of 0.8 µg/mL atc for NadE-DUC and 2 µg/mL atc for PpnK-DUC. At the indicated times protein extracts were prepared and analyzed by immunoblotting. NadE and PpnK were quantified densitometrically and normalized to the no atc controls. **b,** Extent of NadE depletion. NadE was rapidly depleted and below the limit of detection after 6 days, which corresponds to less than 2% of the NadE amount in untreated H37Rv. **c,** Extent of PpnK depletion. PpnK was rapidly depleted and below the limit of detection after 6 days, which corresponds to less than 2% of the PpnK amount in untreated H37Rv.

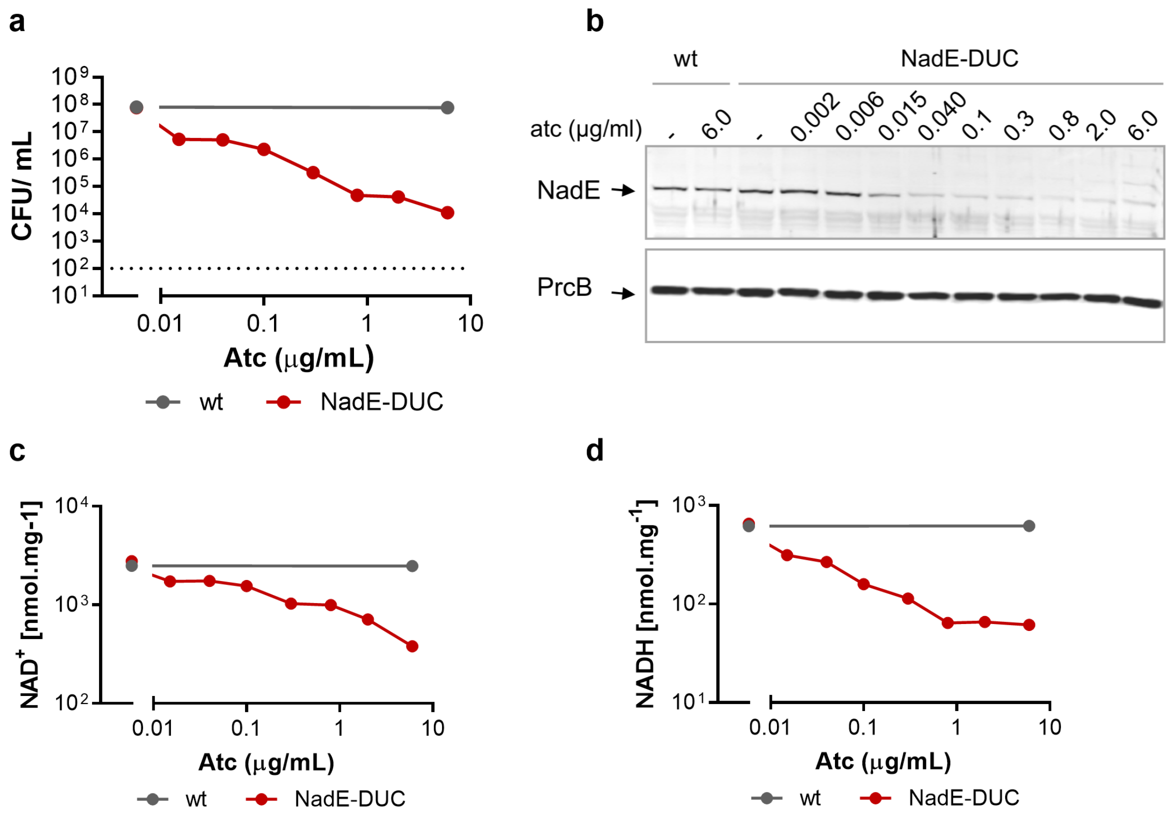

Supplementary Fig 4. Atc dose-responsiveness of NadE-DUC. a, Survival. NadE-DUC was grown on filters in 7H9 containing glucose as the primary carbon source and subjected to varying concentrations of atc. At day 6, CFUs were analyzed by harvesting filters and plating them on 7H11 plates. Dashed lines represent the limit of detection. Data are means ± SD for 4 filters and representative of 2 independent experiments. Error bars are too small to extend beyond the symbols. **b,** Depletion of NadE. Immunoblot of NadE (upper) and loading control PrcB (lower) in extracts isolated from the filters shown in A. **c,** Depletion of NAD^+. Intracellular NAD^+ from H37Rv (grey) and NadE-DUC (red), grown on filters in 7H9 with glucose as the sole carbon source. Data are means ± SD for 3 filters. Data are representative of 2 independent experiments. **d,** Depletion of NADH. Intracellular NADH from H37Rv (grey) and NadE-DUC (red) grown on filters in 7H9 with glucose as the sole carbon source. Data are means ± SD for 3 filters. Data are representative of 2 independent experiments.

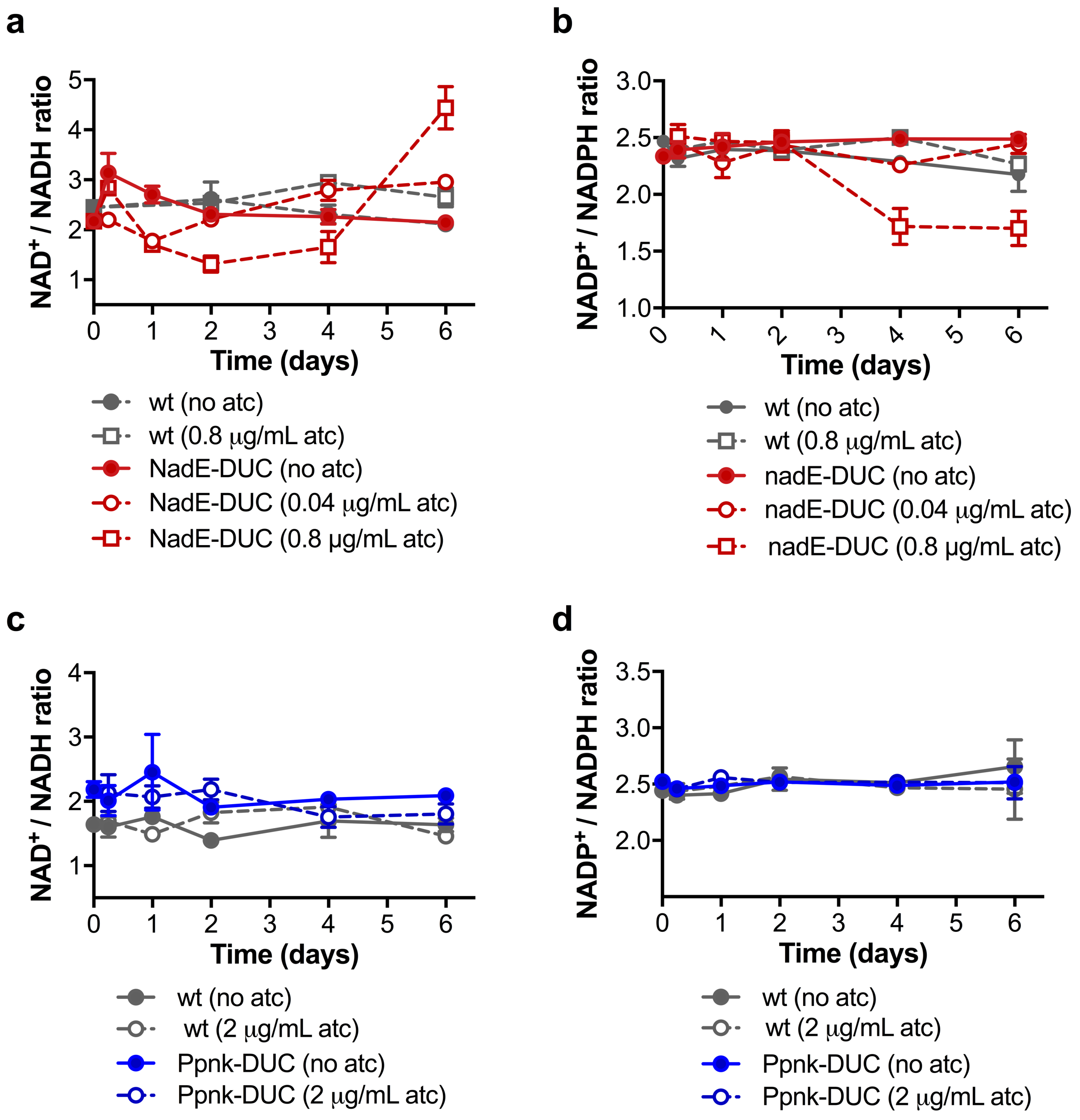

Supplementary Fig. 5. Cofactor ratios upon depletion of NadE or PpnK. NAD^+/NADH ratios (**a** and **c**) and $NADP^+$/NADPH ratios (**b** and **d**) from wt (grey), NadE-DUC (red), or PpnK-DUC (blue) grown on filters in 7H9 with glucose as the sole carbon source. Data are means ± SD for 3 (a) or 6 (b,c,d) filters and representative of at least 2 independent experiments.

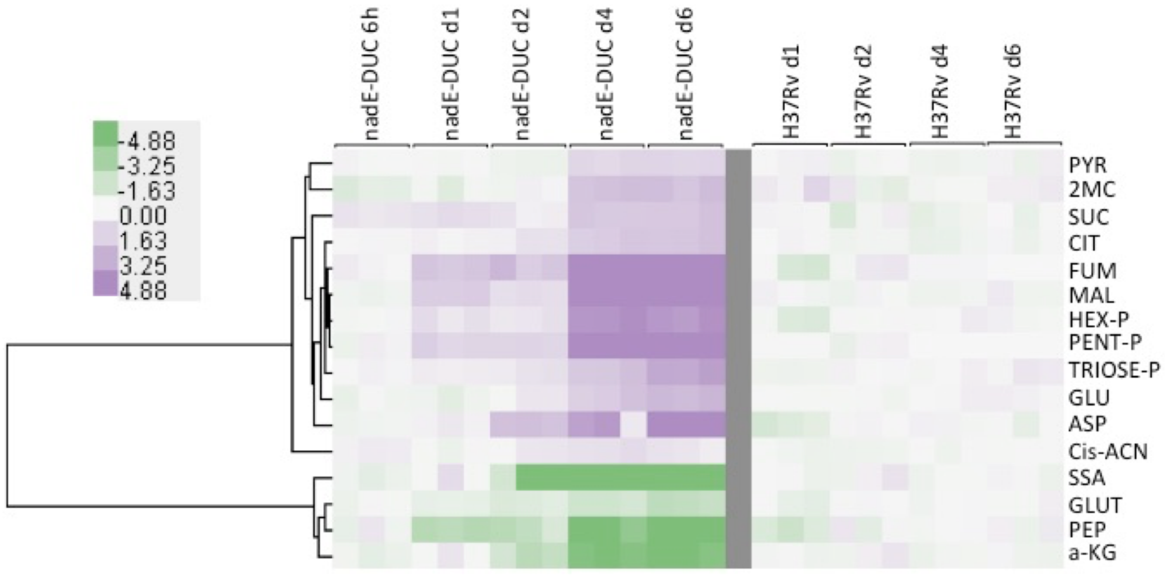

Supplementary Fig. 6. Kinetics of metabolite changes in the CCM pathway upon NadE depletion. Columns depict time points as indicated; rows indicate individual metabolites. Data were parsed using uncentered Pearson's correlation with centroid linkage clustering and rendered using the image generation program treeview (http://treeview.sourceforge.net). Data are on a $\log_2$ scale relative to no atc control at each time point (PYR: Pyruvate, 2MC: 2 Methyl Citrate, SUC: Succinate, CIT: Citrate, FUM: Fumarate, MAL: Malate; HEX-P: Hexose Phosphates, PENT-P: Pentose Phosphates, TRIOSE-P: Triose Phosphates, GLU: Glutamate, ASP: Aspartate, cis-CAN: cis-Aconitate, SSA: Succinic Semialdehyde, PEP: Phosphoenolpyruvate, α-KG: α-Ketoglutarate)

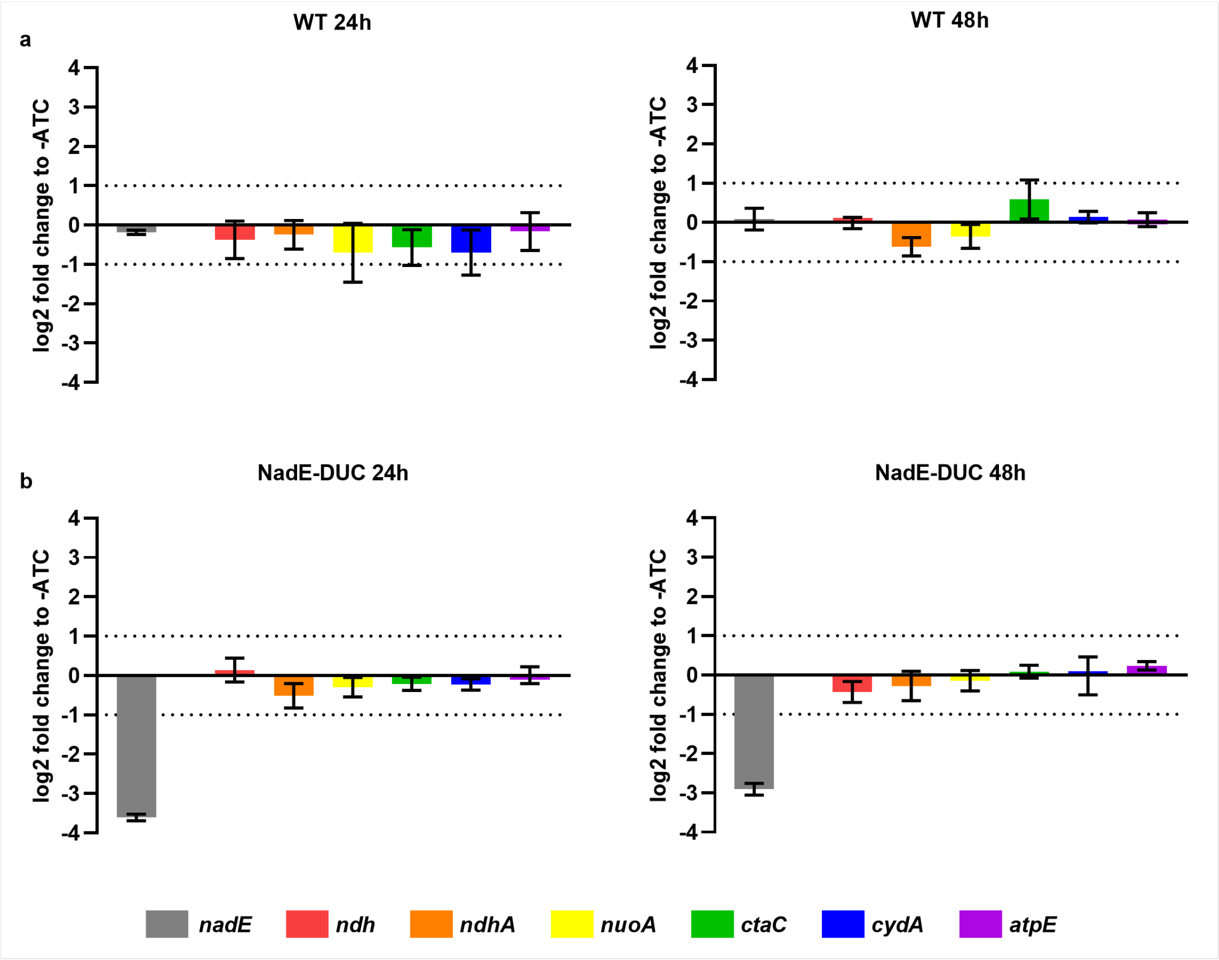

Supplementary Fig. 7. Transcription profile of respiratory chain related genes in response to partial depletion of NadE. *Mtb* H37Rv (**a**) and NadE-DUC (**b**) were grown in 7H9 liquid medium until exponential phase (OD_{580nm} 0.5) and then exposed to atc (0.8 mg/ml) for 24 hours or 48 hours. *sigA* was used as reference gene. qPCR data is represented as log2 fold change relative to no atc condition. Values are the mean of 3 independent experiments. Error bars correspond to standard deviation.

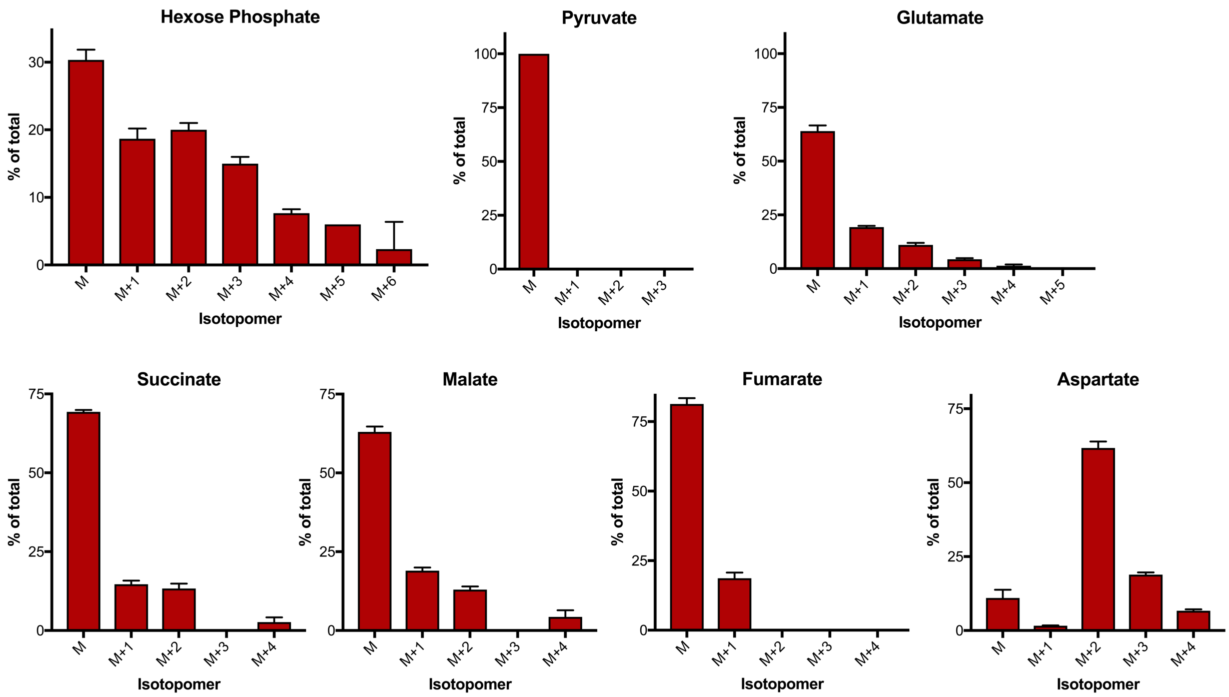

Supplementary Fig. 8. Impact of depleting NadE on the isotopomer profiles of selected metabolites. Isotopomer profiles of metabolites after atc exposure of NadE-DUC for 6 days. Data are means ± SD for 3 filters and representative of 2 independent experiments.

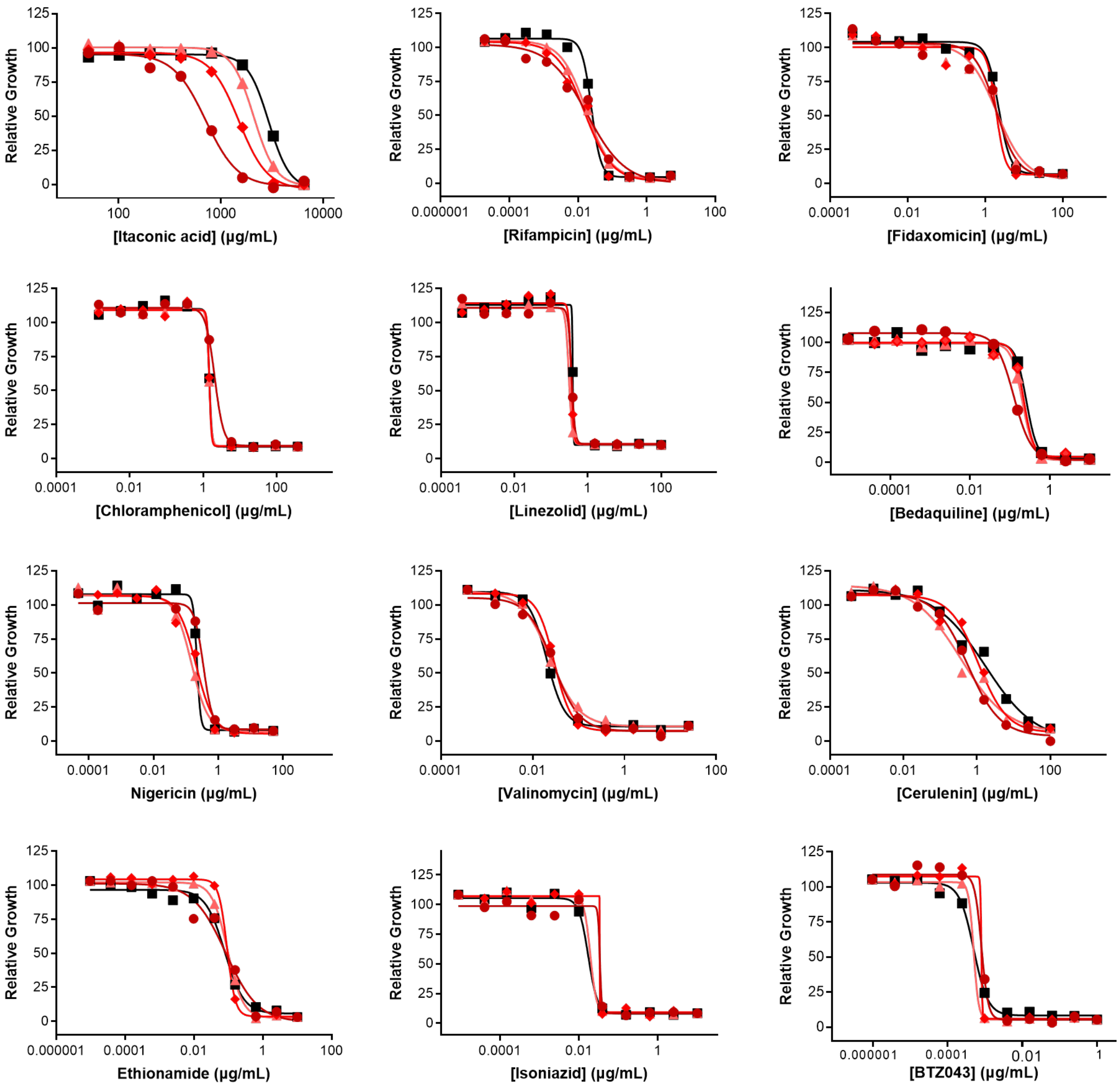

Supplementary Fig. 9. Impact of partial depletion of NadE on the activity of itaconic acid and various drugs. Growth of NadE-DUC was analyzed without atc (black squares) and three different concentrations of atc (low (red triangles, 100 or 125 ng/mL atc), medium (red diamonds, 200 or 250 ng/mL atc), or high (red circles, 400 or 500 ng/mL atc)). Data are averages of at least two cultures per concentration and representative of 2 independent experiments.

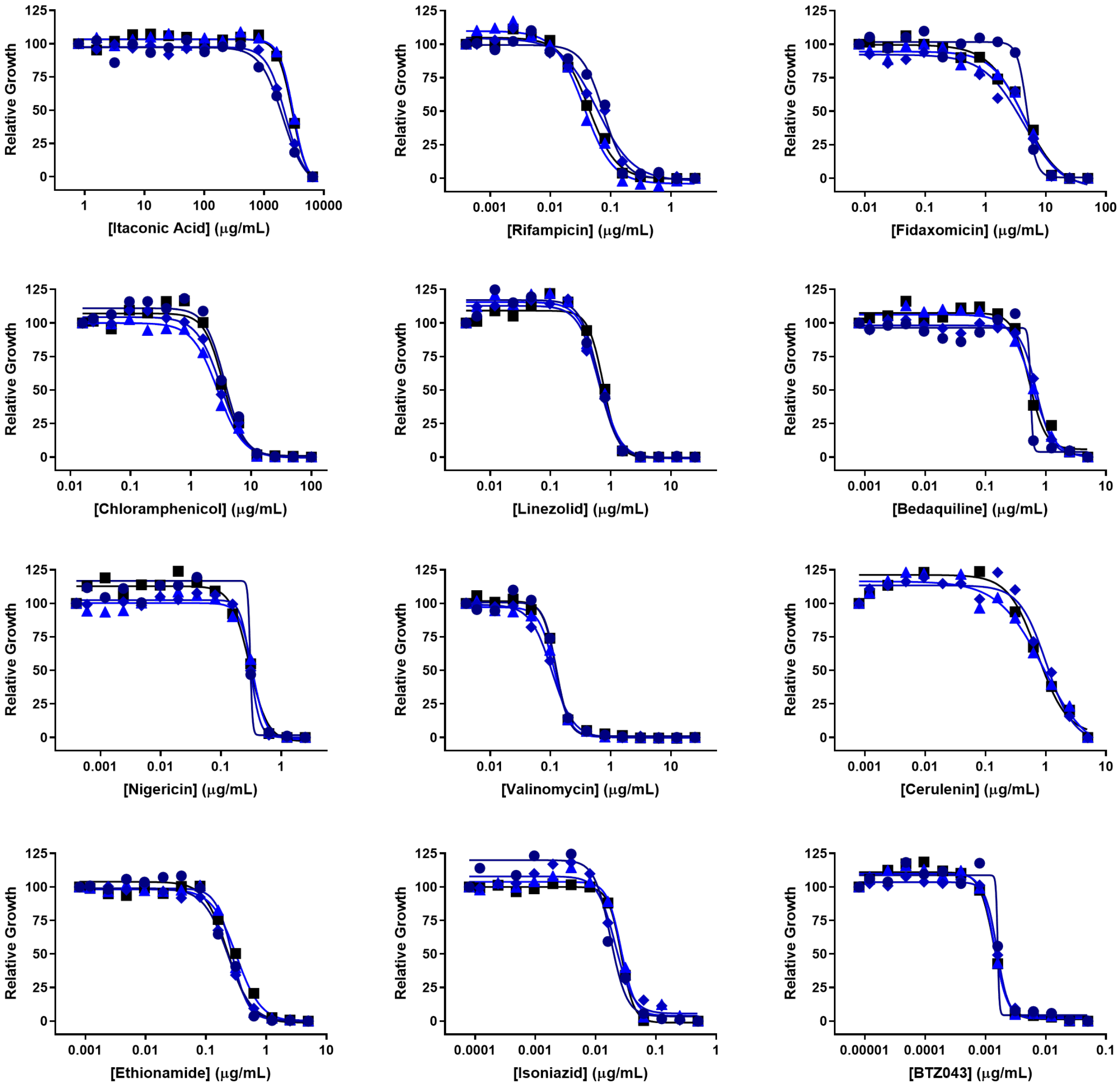

Supplementary Fig. 10. Impact of partial depletion of PpnK on the activity of itaconic acid and various drugs. Growth of PpnK-DUC was analyzed without atc (black squares) and two different concentrations of atc: (blue triangles, 156 ng/mL atc (blue triangles) and 624 ng/mL atc (blue diamonds). Data are averages of at least two cultures per concentration and representative of 2 independent experiments.
